# Conditions of the Stepwise Cooling Algorithm for Stable Supercooling Preservation and Freshness of Pork Loin

**DOI:** 10.3390/foods11244021

**Published:** 2022-12-13

**Authors:** Dong Hyeon Park, Eun Jeong Kim, Honggyun Kim, Geun-Pyo Hong, Mi-Jung Choi

**Affiliations:** 1 Department of Food Science and Biotechnology of Animal Resources, Konkuk University, Seoul 05029, Republic of Korea; 2 Refrigerator Research of Engineering Division, Home Appliance and Air Solution Company, LG Electronics, Changwon 51533, Republic of Korea; 3 Department of Food Science and Biotechnology, Sejong University, Seoul 05006, Republic of Korea

**Keywords:** supercooling, external factor, stepwise algorithm, freshness, pork loins

## Abstract

Supercooling has the advantage of maintaining the freshness of foods without a phase transition. However, it is hard to sustain the supercooled state. Static temperature control, one of the various supercooling technologies, is used for stable supercooling storage. In this experiment, the effect of following external factors in maintaining the supercooled state of foods was investigated. Three main parameters had an effect on the supercooled state of food: (1) properly setting the lower-temperature limit of the supercooling algorithm, (2) slow cooling to the target temperature, and (3) minimizing temperature fluctuation. Accordingly, the following stepwise cooling algorithm for pork loin was designed: a lower-temperature limit of −3.0 °C and a storage period = 36 h followed by a lower-temperature limit of −3.5 °C for 24 h. The samples conserved at −3.0 °C displayed a 100% supercooled state. Physicochemical properties including drip loss, cooking loss, texture, color, total volatile basic nitrogen (TVBN), and total aerobic count (TAC) of pork loin were analyzed. The drip loss values of the supercooled meat samples were lower than those of the superchilled ones. Furthermore, TVBN and TAC of the treated samples were not significantly different from those of the fresh samples (*p* > 0.05). In conclusion, supercooling storage extended the freshness and quality of pork loin better than refrigerated storage.

## 1. Introduction

Most foods easily deteriorate. Therefore, low-temperature storage techniques, including refrigeration and freezing, are used to prolong shelf life. Refrigeration extends the shelf life of food by several days by removing heat [1]. However, this storage method is difficult to preserve long-term due to the deterioration of food quality caused by biochemical and microbial reactions. Freezing is a better way to sustain food freshness than refrigeration for long-term storage. Owing to developing device technologies such as compressor capacity increase, domestic equipment in East Asian countries such as Korea and Japan include deep freezers. Freezing extends the shelf life of food by more than one year. However, ice crystal formation in frozen foods [2] and ice recrystallization over long storage periods cause quality degradation resulting in drip loss after thawing [3,4].

Researchers and manufacturers have been interested in superchilling and supercooling as novel storage technologies. Superchilling lowers the freezing point of food stuff by 1.0–2.0 °C resulting in the freezing of approximately 30–50% of the water in food [5]. However, this technique may lead to quality loss generated by ice crystals [6]. Supercooling lowers the temperature of food below its freezing point, without water-to-ice transition [4]. This novel technique has the potential to extend the shelf life of food without destroying tissue quality by preventing ice crystal formation [3]. However, the supercooled state is metastable, and the unstable arrangement of water molecules can lead to ice nucleation at any time if an external force such as vibration is applied [7]. To overcome the metastable supercooling condition, advanced technologies such as high pressure, electric and magnetic fields, and ultrasonic vibrations have been investigated for application in food systems [7,8,9,10]. However, limited techniques are available for extending the duration of supercooled conditions, with some being costly to apply in the food industry [11].

Static temperature control with a low cooling rate has been suggested for maintaining supercooled conditions. Fish flesh can be preserved without freezing for 9 days at −5.0 °C under static temperature air control [12]. To achieve this, stepwise cooling with a decrease of −0.5 °C every 24 h is applied. Slow cooling effectively maintains a supercooled state for approximately 3–4 h [13,14]. These studies concentrated on maximizing the degree of supercooling but not on keeping the supercooled condition stable during the storage period. Recently, supercooling preservation experiments using a stepwise cooling algorithm were conducted [3,7]. This method applies regular temperature-decrease cycles to prevent food from freezing. Furthermore, since the optimal preservation conditions for each food are different, it is necessary to identify and set accurate food-specific temperature-control programs. In this experiment, we investigated the effect of external factors on cooling media temperature control to sustain the supercooled state of food and analyzed the freshness of pork loin under different preservation conditions.

## 2. Materials and Methods

### 2.1. Sample Preparations

Pork loins, purchased 48 h post-mortem from a local mart, were cut into 30 × 30 × 30 mm^3^ (width × length × height) cubic shapes. Subsequently, the meat was wrapped twice in plastic wrap. T-type thermocouples were attached to the surface to monitor temperature variations, and the temperature was recorded every 1 min during storage. A data logger (Data Acquisition MX-100, Yokogawa, Tokyo, Japan) was used to investigate whether the temperature of the samples was suitable and whether the samples needed to be frozen. Untreated meat samples were used as a control.

### 2.2. Temperature Control

#### 2.2.1. Limit of Lowest Temperature

A total of 32 samples were prepared and divided into 4 groups. Eight samples were placed in separate polypropylene boxes and arranged in 2 rows of 4. Each box was placed in a separate refrigerator (K417SS13, LG Electronics, Seoul, Republic of Korea) set at −1.0–−4.0 °C (at 1.0 °C intervals) and stored for 5 days. The limit of lowest temperature was defined when the storage temperature was at its lowest.

#### 2.2.2. Effect of Slow Cooling on the Supercooled State

##### Supercooling Stability of Pork Loin by Different Cooling Rates

Sixteen pork loins were placed in separate polypropylene boxes and arranged in 2 rows of 4. The boxes were stored in two refrigerators (K417SS13; LG Electronics, Seoul, Republic of Korea). Based on the result of the previous step, the target temperature was set at −4.0 °C to freeze the samples, and the cooling rate was divided into two treatments. The temperature of one refrigerator was decreased by 0.5 °C every 80 min, and that of the other was reduced by 0.5 °C every 4 h.

##### Cooling Rate Control Using a Stepwise Cooling Algorithm

A total of 32 samples of meat were prepared and divided into two groups. Sixteen samples were arranged in 4 rows of 4 and stored in refrigerators (K417SS13, LG Electronics) set at −3.0 and −4.0 °C. To attain the target temperature of −3.0 °C through slow cooling, the initial temperature was set at −0.5 °C and reduced by 0.5 °C every 80 min or 4 h. The target temperature of −4.0 °C was achieved similarly; however, after reaching −3.0 °C, the temperature was drastically lowered to −4.0 °C. All samples were stored for 168 h at the target temperature.

##### Stepwise Cooling Algorithm for the Supercooling Storage of Pork Loin

Twenty-four pork loins were allocated to two polypropylene boxes and arranged in 4 rows of 3. Each box was preserved in six refrigerators (K417SS13, LG Electronics). To apply slow cooling for supercooling storage, the initial temperature of all the refrigerators was set at −1.0 °C for 24 h and reduced by 0.5 °C every 4 or 8 h. The temperature was maintained after reaching −3.0, −3.5, and −4.0 °C for 96 h. After that, the temperature was increased to −1.0°C to inhibit ice nucleation and then decreased by 0.5 °C every 4 or 8 h. This temperature-control program was repeated every 96 h to prevent ice nucleation.

#### 2.2.3. Effect of Temperature Fluctuations on the Supercooled State

**Figure 1:**
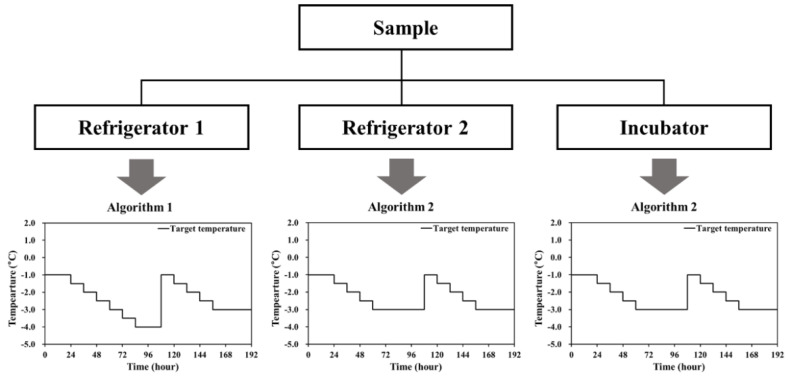
Experimental protocol for temperature fluctuation.

To confirm the effect of temperature fluctuations on maintaining the supercooled state of the pork loins, 54 meat samples were prepared. Eighteen loins were placed in two polystyrene boxes (20 mm thickness) and arranged in 3 rows of 3. Each box was stored in a refrigerator (K417SS13, LG Electronics) or incubator (FMU-0531, Fukushima Industries Corp., Osaka, Japan), adopting one of the two supercooling algorithms. The first cooling program was initially set at −1.0 °C for 24 h and reduced by 0.5 °C every 12 h. The lowest temperature was maintained at −4.0 °C for 24 h. The temperature was then restored to −1.0 °C and again decreased by 0.5 °C every 12 h. After reaching −3.0 °C, the temperature was maintained until the end of the experiment. The initial temperature of the second algorithm was also −1.0 °C; the temperature was decreased by 0.5 °C every 12 h until reaching the lower-temperature limit set to −3.0 °C, which was maintained for 48 h. After raising the temperature to −1.0 °C, this algorithm cycle was repeated. The first and second cooling algorithms were applied for the refrigerators, whereas only the second was applied for incubators (Figure 1).

### 2.3. Characteristics of Food Freshness

#### 2.3.1. Condition for Stepwise Cooling

Preliminary experiments showed that stepwise cooling for supercooled storage effectively maintained the supercooled state of the pork loin. The supercooling treatment was simultaneously conducted in six refrigerators (K417SS13, LG Electronics) set to a specific stepwise cooling algorithm. A total 114 meat samples were prepared and separated into 4 groups (9 samples × 4 types of preservation × 3 replications). They were placed in polystyrene boxes (20 mm thickness) and arranged in 3 rows of 3. Each box was stored in a refrigerator. The initial temperature was set at −1.0 °C and decreased 4–5 times by 0.5 °C every 12 h. After reaching −3.0 or −3.5 °C, the temperature was maintained for 36 or 24 h, respectively. These stepwise cooling algorithms were repeated every 4 days. To compare the temperature effect between the supercooling and superchilling treatments, a freezing treatment was performed at −3.0 °C. Samples were initially superchilled at −5.0 °C and then stored in a refrigerator (K417SS13, LG Electronics) set at −3.0 °C. Each polystyrene box containing eight samples was refrigerated (R-F875HBSW, LG Electronics) at a temperature set to 2.0 °C. The boxes were removed and the samples unpacked for physicochemical analysis after eight days. Superchilled samples were thawed at 3.0 °C until the core temperature reached 0 °C. The remaining 6 samples were used as controls.

#### 2.3.2. Drip Loss

Before preservation, fresh meats were weighed. After storage, the moisture of each treated sample was removed using a tissue and then weighed again. Drip loss was expressed as the percentage ratio of the removed weight to the initial weight of the sample, as follows:

(1) $$Drip\;loss\;\left(\%\right)=\frac{W_{1}-W_{2}}{W_{1}}\times 100$$

where *W*_1_ was the initial weight of the sample (g), and *W*_2_ was the weight of the treated and wiped sample (g).

#### 2.3.3. Cooking Loss

The cooking loss of samples was determined using a procedure conducted by Kim [7]. The samples were placed into plastic bags and vacuum-sealed. After that, they were heated in a water bath (BF-30SB, BioFree, Seoul, Republic of Korea) at 70 °C for 30 min. The heated samples were then cooled to room temperature (25 °C) for 30 min. The samples were weighed before and after cooking. The cooking loss of meat samples was calculated using the following formula:

(2) $$Cooking\;loss\;\left(\%\right)=\frac{W_{3}-W_{4}}{W_{3}}\times 100,$$

where *W*_3_ was the weight of the fresh sample (g) and *W*_4_ was the weight of the cooked sample (g).

#### 2.3.4. Texture profile analysis

Fresh and treated pork loin samples for which cooking loss was measured were cut into 10 × 10 × 10 (width × length × height) mm^3^ cubes. Texture profile analysis (TPA) was performed using a CT3 texture analyzer (AMETEK Brookfield, Middleboro, MA, USA). The test parameters were set as follows: TA3/100 probe, TA/SBA fixture, test speed of 2 mm/s, trigger load of 5 g, and compression of 70% (Choi et al., 2018).

#### 2.3.5. Color

Color parameters were performed using a colorimeter (CR-400 Chroma Meter; Konica Minolta Sensing, Inc., Tokyo, Japan). The results were expressed as lightness (Commission Internationale de L’eclairage (CIE) L*), redness (CIE a*), and yellowness (CIE b*). The values of the meat samples were measured after calibration using a white standard plate (CIE L* = 96.79, CIE a* = 0.30, and CIE b* = 1.67). The total color difference (ΔE) was calculated using the following equation to determine the differences between the fresh and treated pork loin samples:

(3) $$\Delta E=\sqrt{{\left({\Delta \mathrm{CIE}\;L}^{*}\right)}^{2}+{\left({\Delta \mathrm{CIE}\;a}^{*}\right)}^{2}+{\left({\Delta \;\mathrm{CIE}\;b}^{*}\right)}^{2}}$$

#### 2.3.6. Total Volatile Basic Nitrogen

The total volatile basic nitrogen (TVBN) value of pork loin samples was determined using Conway’s micro-diffusion method [15]. Five grams of meat was homogenized with 45 mL of distilled water using a slap-type homogenizer (WS-400, Shanghai Zhisun Equipment Co., Ltd., Shanghai, China) for 180 s. The homogenate was filtered using Whatman No. 2 filter paper (GE Healthcare, Chicago, IL, USA). After filtration, 1 mL of filtrate was placed on the outer side of a Conway dish containing 1 mL of 0.01 N H_3_BO_3_ mixture, and 100 μL of Conway solution (0.066% methyl red in ethanol: 0.066% bromocresol green in ethanol) was dropped into the inner side of the Conway dish. Next, 1 mL 50% K_2_CO_3_ was added to the outer side of the dish. The lid of the Conway dish was closed, and the dish was incubated at 37 °C for 2 h in a thermo-hygrostat (IL3-25A, JEIO Tech, Daejeon, Republic of Korea). The TVBN content was determined following the addition of 0.02 N H_2_SO_4_ to the inner side of the Conway dish. A blank test was performed following the same process without the addition of the homogenized samples. The TVBN value was calculated as follows:

(4) $$\mathrm{TVBN}\;\left(\mathrm{mg}/100\;g\right)=\frac{14.007\times \left(a-b\right)\times f\times 100\times c}{S},$$

where *a* is the titration volume of the sample (mL), *b* is the titration volume of the blank (mL), *f* is the factor of H_2_SO_4_, *S* is the weight of the sample, and *c* is the dilution factor.

#### 2.3.7. Microbial Analysis

Two grams of each treated sample was separated from the surface and homogenized with 9 mL of sterilized 0.85% NaCl solution for 1 min using a slap-type homogenizer (WS-400; Shanghai Zhisun Equipment Co., Ltd., Shanghai, China). The supernatant was serially diluted, and 1 mL of the diluted solution was placed on ready-to-use Petrifilm aerobic count plates (3M Petrifilm^TM^, 3M, Maplewood, MN, USA) and incubated for 2 d at 37 °C in an incubator (SI-600R, Jeio Tech, Seoul, Republic of Korea). The number of colonies in the samples was expressed as a logarithm of the number of colony-forming units in 1 g of the sample (log CFU/g).

#### 2.3.8. Statistical Analysis

All experiments were repeated three times for replication. Data are expressed as the mean ± standard deviation. One-way analysis of variance (ANOVA) was performed, and mean comparisons were determined using Duncan’s multiple range test (*p* < 0.05).

## 3. Results and Discussion

### 3.1. Limit of Lowest Temperature

**Figure 2:**
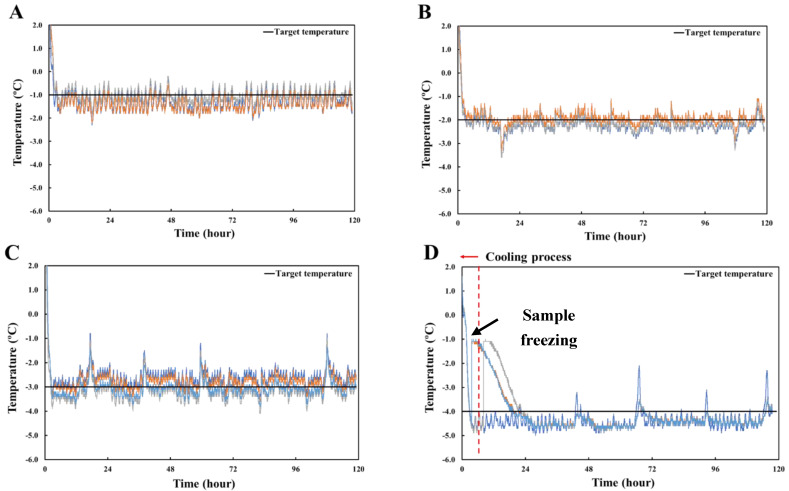
Time−temperature profiles of target temperature and pork loin with different sub-zero storage temperatures for 120 h. (**A**) –1.0 °C; (**B**) –2.0 °C; (**C**) –3.0 °C; (**D**) –4.0 °C. The color of the line in the figure indicates the sample to which the thermocouple is connected.

The changes of temperature for pork loin samples at different subzero-preserving temperatures are presented in Figure 2. The storage temperature of samples treated at −1.0 °C was kept stable (Figure 2A), and their temperatures were 0.3 °C lower than the target temperature. The average temperature of the samples was −1.1 °C, and none of the meat samples were frozen during the storage period. The temperature of the samples stored at −2.0 °C was also stably controlled, and there was a slight difference between the target temperatures (Figure 2B). The average temperature of the meat samples was −2.0 °C, and the samples remained unfrozen during storage. The temperature of samples preserved at −3.0 °C was relatively unstable compared to those stored at −1.0 and −2.0 °C, indicating increased temperature fluctuation (Figure 2C). Despite this instability, the samples remained unfrozen. However, the temperature of the samples stored at −4.0 °C fluctuated, and ice nucleation was detected during the storage period. Most ice nucleation occurred during the cooling process, and the ice nucleation temperature of the samples was approximately −4.5 °C. Based on these results, supercooling preservation of pork loin up to −3.0 °C was successful for 120 h with no failures for samples stored below −0.6 °C, which is the initial freezing-point temperature for pork loin [13]. The nucleation temperature generated a relatively rapid decrease. Therefore, slow cooling is required to achieve stable supercooling storage [16].

### 3.2. Effect of Slow Cooling on the Supercooled State

#### 3.2.1. Supercooling Stability of Pork Loin Stored at Different Cooling Rate

**Figure 3:**
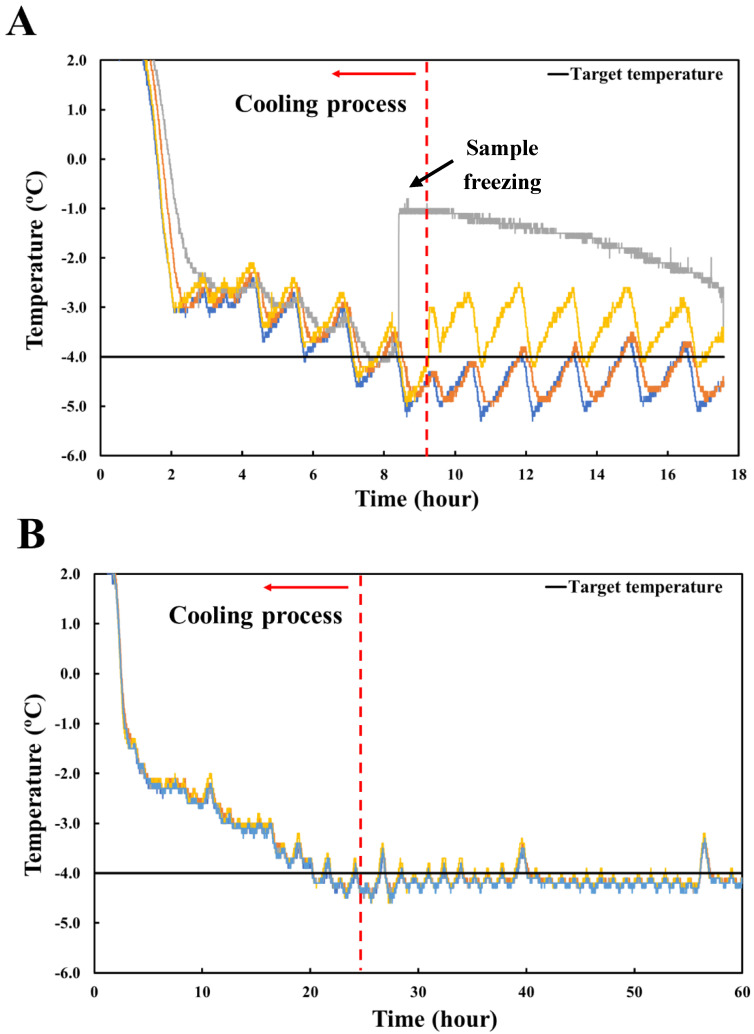
Time−temperature profiles of target temperature and pork loin stored with different cooling rates. (**A**) Decreased by 0.5 °C every 80 min; (**B**) decreased by 0.5 °C every 4 h. The color of the line in the figure indicates the sample to which the thermocouple is connected.

The temperature curves and the target temperature of pork loin stored at different cooling rates are shown in Figure 3. The temperature of the samples was rapidly cooled to −2.0 °C within 2 h, and then decreased by 0.5 °C every 80 min (Figure 3A). Furthermore, it took approximately 9.2 h to reach the target temperature of −4.0 °C for the samples; the cooling rate was −0.98 °C/h. The temperature deviation was more than ± 0.5 °C at the ending temperature, and the samples froze during the cooling process. During storage, the meat samples were not frozen. The temperature of the samples increased by more than 1.0 °C as the thermocouple was disconnected from the samples. The temperature deviation was also increased by approximately 0.05 °C. In contrast, the temperatures of the other treatments were slowly cooled down to −2.0 °C for about 4.5 h and reduced by 0.5 °C every 4 h (Figure 3B). The samples were cooled for 24 h at a cooling rate of −0.38 °C/h. Compared to the 80 min reduction treatment, the cooling time was more than doubled, and the cooling rate was lowered more than twofold. In addition, it was effective in keeping chicken breasts in a supercooled state at −2 °C using a slow cooling rate [7]. Nevertheless, the samples remained unfrozen during cooling and storage, indicating that a low cooling rate is more effective than a high cooling rate in lowering the temperature of the pork loin.

#### 3.2.2. Cooling Rate Control Using a Stepwise Cooling Algorithm

**Figure 4:**
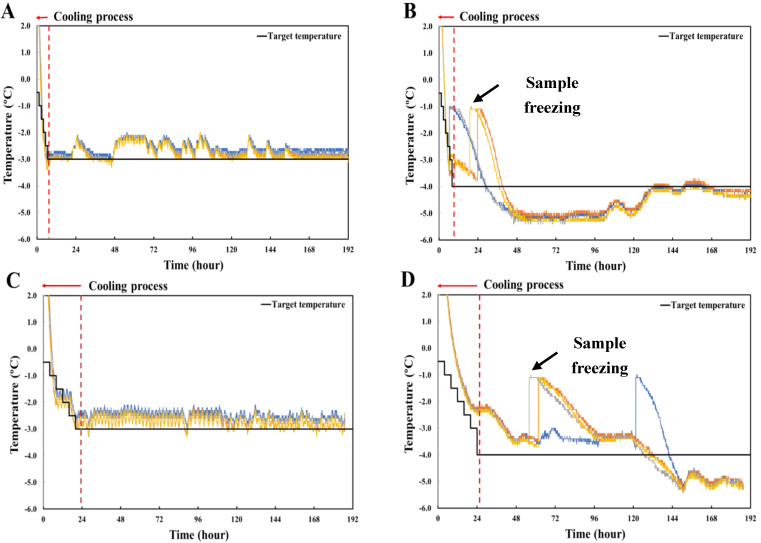
Time–temperature profiles of target temperature and pork loin stored with stepwise cooling for 192 h. (**A**,**B**) Decreased by 0.5 °C every 80 min; (**C**,**D**) decreased by 0.5 °C every 4 h. The color of the line in the figure indicates the sample to which the thermocouple is connected.

The time–temperature profiles and the target temperature of pork loin stored with stepwise cooling for 192 h are shown in Figure 4. Based on the results of the previous step, stepwise cooling was adopted to achieve a low cooling rate. The cooling rates applied to attain the target temperature were −0.63 °C/h (Figure 4A) and −0.33 °C/h (Figure 4B). In addition, the cooling rates for the target temperature of −4.0 °C were −1.04 °C/h (Figure 4C) and −0.32 °C/h (Figure 3D). All the samples cooled to the target temperature of −3.0 °C were stable and remained unfrozen. In contrast, the cooling rate displayed in Figure 4D was the highest among all treatments; half of the samples demonstrated a phased transition during the cooling process at a nucleation temperature of approximately −3.5 °C. The remaining samples were frozen during storage. After storage, the temperatures of the samples were lower than the target temperature for 36–132 h. The cooling rate shown in Figure 4D was the lowest among all treatments. Although the samples did not freeze during the cooling process, they froze after reaching the lower-temperature limit of the treatment. The ice nucleation temperature was approximately −3.6 °C. This result demonstrates that stepwise cooling effectively prevents phase transitions during freezing [3,7,16]. Particularly, the longer the time taken to attain the lower-temperature limit of a treatment, the more stable the decrease in food temperature [7]. However, food is considered frozen during the storage period because it is relatively unstable at lower temperatures. Therefore, it is necessary to recover the initial phase temperature to prevent ice nucleation.

#### 3.2.3. Stepwise Cooling Algorithm for Supercooling Storage of Pork Loin

**Figure 5:**
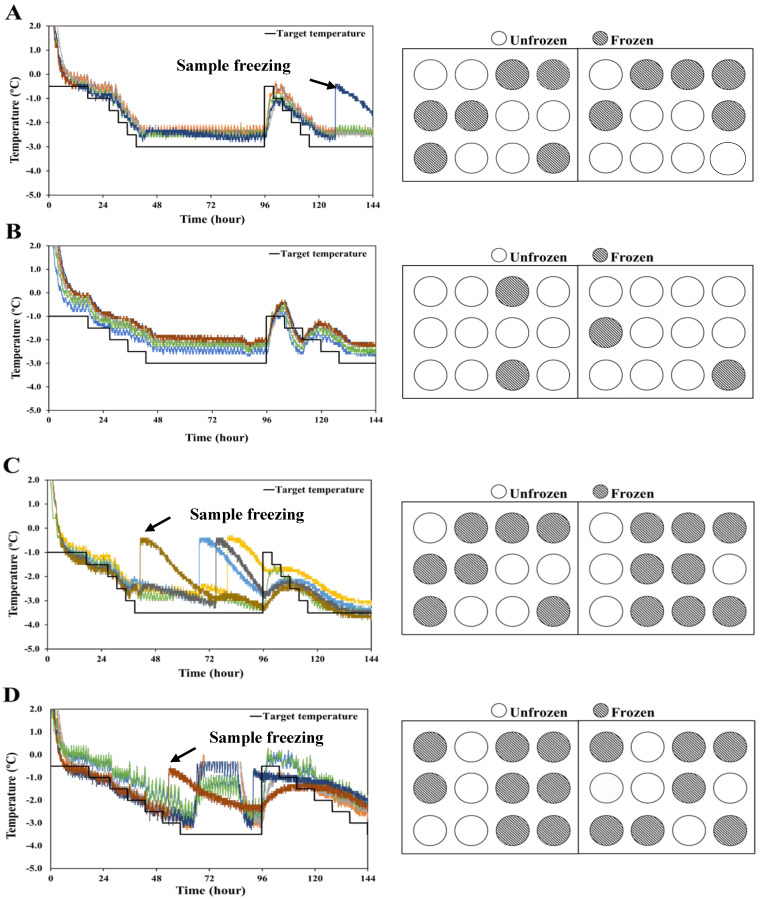

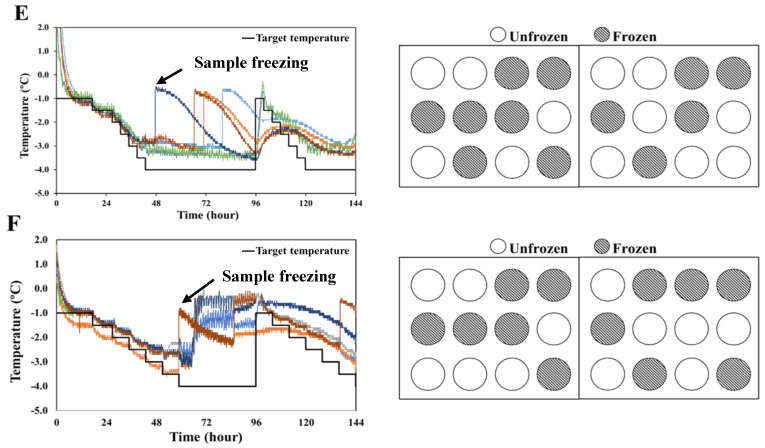
Time–temperature profiles of supercooled storage applied using various stepwise cooling algorithms for slow rate (left side) and layout of supercooled state (right side) during storage periods. (**A**) Decreased by −0.07 °C/h for −3 °C; (**B**) decreased by −0.04 °C/h for −3 °C; (**C**) decreased by 0.07 °C/h for −3.5 °C; (**D**) decreased by −0.04 °C/h for −3.5 °C; (**E**) decreased by −0.07 °C/h for −4 °C; (**F**) decreased by −0.04 °C/h for −4 °C. The color of the line in the figure indicates the sample to which the thermocouple is connected.

The experimental design implemented for various treatments is presented in Table 1. To achieve a low cooling rate for the supercooled preservation of meat, a stepwise cooling algorithm was adopted. Once the target lower-temperature limit of the treatments was reached, the temperature was raised to the initial temperature of −0.5 or −1.0 °C to prevent ice nucleation. The time–temperature profiles of the supercooled storage periods applied with slow-rate stepwise cooling to the supercooled state are shown in Figure 5. The temperatures of treatments A and B were regulated to be higher than the target temperature. Of the twenty-four samples, eleven and four were frozen during storage in treatments A and B, respectively; thus, approximately 54.2% and 83.3% of the samples, respectively, sustained supercooled conditions. The temperature of the pork loin samples for treatments C and D followed the target temperature. During the control of cooling rates of the samples using two different algorithms, 15 of the 24 samples were frozen, while the remaining 37.5% of the samples was maintained in a supercooled state. The temperatures of the samples for treatments E and F were slightly higher than the target temperature. During supercooled storage of the samples with treatments E and F, 12 of the 24 samples remained unfrozen; thus, 50% of the samples remained in the supercooled state. As the lower temperature limit was below −3.5 °C and the cooling time interval was shorter, many samples were frozen. According to Kim [7] and Park et al. [3], meat and fish samples stored in a supercooling condition with a stepwise cooling algorithm were stably preserved for two weeks. Considering these results, it can be assumed that slow stepwise cooling is important for maintaining the supercooled condition of pork loins. Furthermore, the time intervals and lower temperature limits of the treatments were significant factors for the success of maintaining the supercooled state.

### 3.3. Effect of Temperature Fluctuations on the Supercooled State

**Figure 6:**
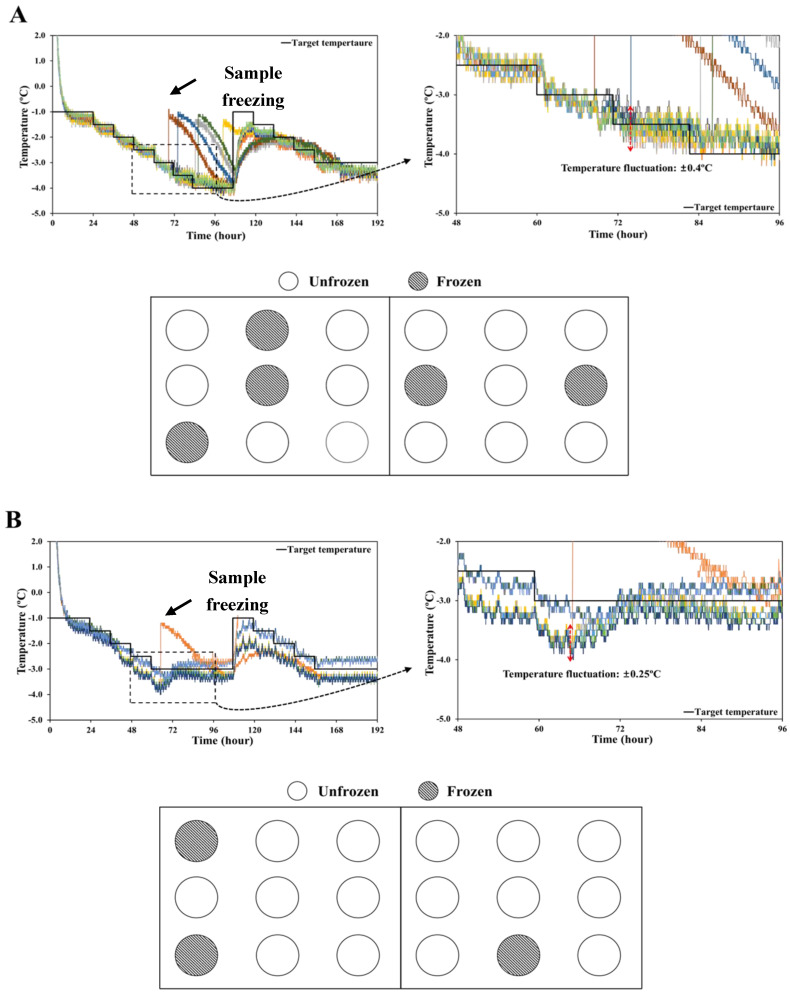

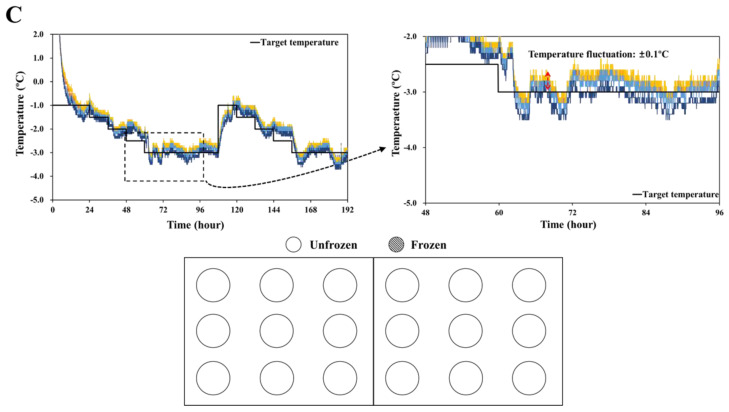
Time−temperature profiles of supercooled storage preserved in a refrigerator (**upper**), and layout of supercooled state (**below**) during storage periods. (**A**) Refrigerator adopted for algorithm 1; (**B**) Refrigerator adopted for algorithm 2; (**C**) Incubator adopted for algorithm 2. The color of the line in the figure indicates the sample to which the thermocouple is connected.

The temperature curves for supercooled storage of pork loins and the layout of the supercooled state during various storage periods are shown in Figure 6. The samples stored in refrigerator-1 (Figure 6A) were slowly cooled to the lower-temperature limit. It was observed that many samples were frozen between −3.0 and −4.0 °C, and the temperature deviation was ± 0.4 °C, the highest among all treatments. According to Cho et al. [17], the nucleation temperature of pork loin was approximately −7.6 °C, which was lower than measured temperatures. During storage, out of 18 pork loin samples, 13 remained unfrozen; thus, approximately 72% of the samples sustained a supercooled state. The temperature of pork loins stored in refrigerator-2 (Figure 6B) was approximately 0.3 °C lower than the target temperature. During refrigerator preservation controlled by the second algorithm, one of the 12 samples remained frozen; therefore, approximately 83.3% of the samples remained in a supercooled condition. The temperature fluctuation was ± 0.25 °C, which was lower than that of refrigerator-1 (Figure 6A). The pork loin samples stored in the incubator were cooled down slowly in stages and maintained at −3.0 °C; all samples remained unfrozen. Therefore, this treatment resulted in 100% of the samples being maintained in a supercooled state and had a higher success rate of supercooling than the refrigerator treatments. This result can be explained by the lower temperature fluctuation of the incubator treatments (± 0.1 °C) compared to the ± 0.4 and ± 0.25 °C fluctuations for the refrigerator treatments. Reducing temperature fluctuations is important for preventing ice nucleation during supercooled storage [7]. Furthermore, minimization of temperature deviation is recommended during sub-zero preservation and stages [18]. Hence, it was concluded that the temperature fluctuation should be managed within ± 0.1 °C to increase the success rate of maintaining the supercooled state.

### 3.4. Characteristics of Food Freshness

#### 3.4.1. Time–Temperature Profiles

**Figure 7:**
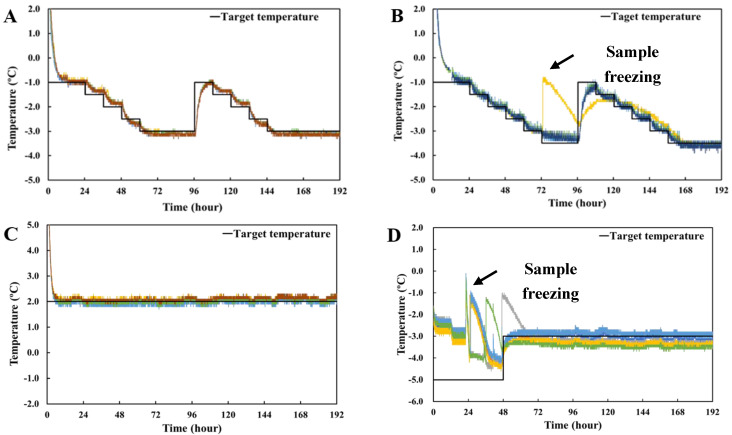
Time−temperature profiles of pork loin during different preservation treatments. (**A**) Supercooling (−3 °C); (**B**) supercooling (−3.5 °C); (**C**) refrigeration; (**D**) superchilling. The color of the line in the figure indicates the sample to which the thermocouple is connected.

The time–temperature profiles for various storage treatments of pork loin and the layout of the supercooled state during the storage period are presented in Figure 7. Based on the results from the previous step, a stepwise cooling algorithm considered three important factors: (1) setting a suitable limit for the lower temperature, (2) minimizing temperature fluctuation (within ±0.2 °C), and (3) returning to the initial temperature of the first cycle at the ending point. Based on these parameters, a supercooling algorithm for pork loin was designed [7]. The optimal supercooling temperature was determined using a stepwise cooling algorithm for pork loin, considering the important external factors for supercooling maintenance. The average target lower-limit temperatures were −2.7 and −2.8 °C, lower than the initial freezing temperature of pork loin [13]. Thus, it was shown that a stepwise cooling algorithm effectively maintained a stable supercooled state of the pork loin. The samples were cooled slowly and maintained at −3.0 and −3.5 °C for 36 and 24 h, respectively. After that, the target temperature was increased to −1.0 °C, and the stepwise cooling process was repeated. All 27 samples maintained the supercooled state for up to 8 days of storage, and ice was not found on the surface of the samples stored at the supercooled treatment of −3.0 °C at the end of storage (Figure 7A). However, the supercooling success rate of samples preserved at −3.5 °C was approximately 93% (25 out of 27 samples), and ice was founded on the surface of the meat samples on the last day of preservation (Figure 7B). Supercooled storage samples were assessed for physicochemical properties, and frozen samples were excluded from this experiment. In the case of refrigeration with storage at 2.0 °C, all the pork loin samples reached 2.0 °C after 2.5 h of storage (Figure 7C). During freezing, the samples were stored at −5.0 °C for 48 h and then preserved at −3.0 °C. All samples started to freeze within 24–48 h, and the freezing point was between −4.1 and −4.5 ºC. The frozen samples remained within the range of −2.7–−3.5 °C (Figure 7D).

Maintaining the supercooled state of foods is difficult because of limited heat conduction and ice crystallization on the surface [16]. Temperature fluctuations and vibrations should be minimized to maintain a supercooled state in solid foods [4]. Therefore, a low cooling rate is essential and an important element for ice nucleation [16,19]. Stepwise cooling for supercooling storage was successful, and samples were maintained in the supercooled state until −5.0 °C or even −7.0 °C [12]. Stepwise cooling effectively retained the supercooled state of fish samples [7,20]. The current study also demonstrates that stepwise cooling is effective to maintain a supercooled state.

#### 3.4.2. Drip Loss and Cooking Loss

**Figure 8:**
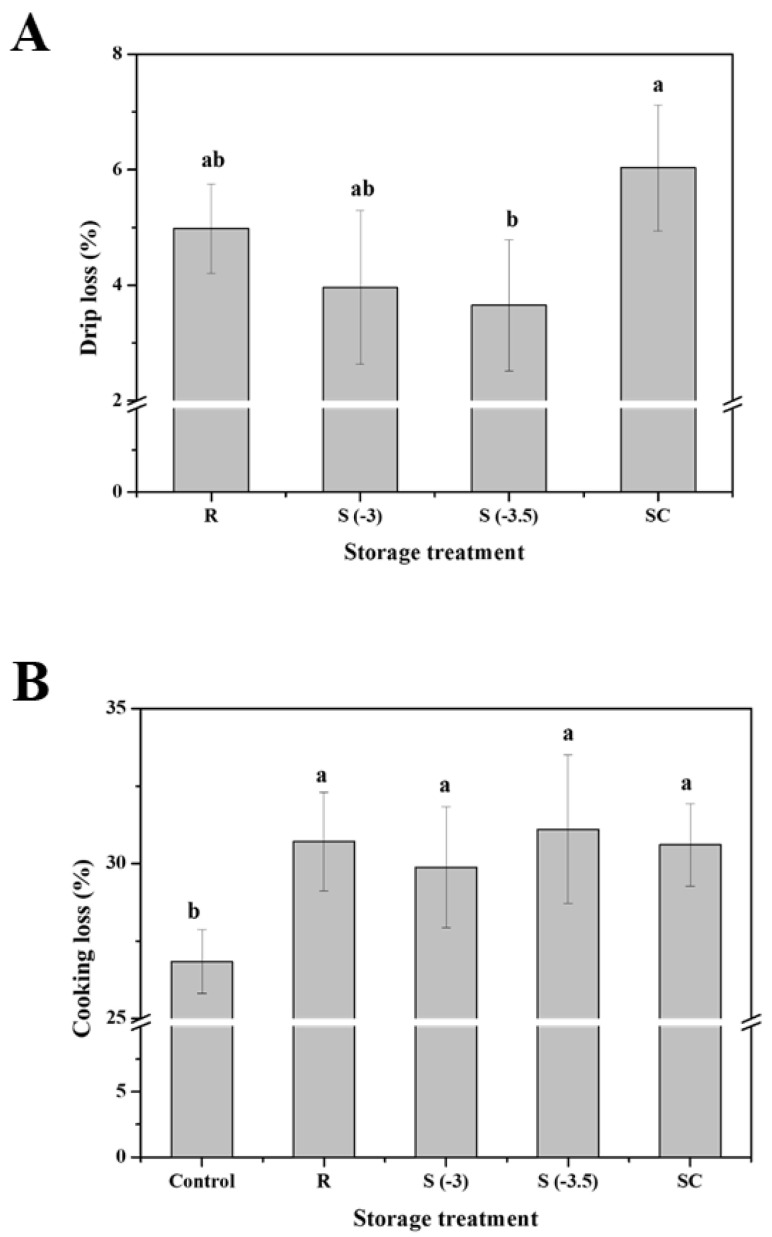
Changes in the (**A**) drip loss and (**B**) cooking loss of pork loin with various preservation treatments. ^a, b^ Indicators using different letters are significantly different (*p* < 0.05). R: Refrigeration; S (−3): Supercooling (−3 °C); S (−3.5): Supercooling (−3.5 °C); SC: Superchilling; Control: fresh meat.

The drip loss of pork loin samples subjected to different storage treatments is shown in Figure 8A. After eight days of storage, the drip loss values of the treated meat samples were 4.98%, 3.96%, 3.65%, and 6.03%. The superchilled samples showed significantly higher drip loss compared to those of the other treatments (*p* < 0.05). The superchilling process produces the greatest drip loss due to ice crystallization [21]. The drip loss of the supercooled samples was significantly similar for the same storage period (*p* > 0.05). However, the drip loss of the samples preserved under refrigeration was higher than those of the supercooled samples; no significant difference was seen among the samples stored at −3.0 °C in supercooled storage (*p* > 0.05). Similarly, the drip loss values of chicken breast samples stored under supercooled conditions were lower than those of samples stored under refrigeration and superchilling conditions [9]. Meat quality deteriorates due to drip loss through weight and nutrient loss [7,22]. Thus, it is essential to rapidly cool the meat to inhibit microbial metabolism [23]. Pork roast preserved at 3.5 °C presented a higher drip loss value than that conserved at −2.0 °C, probably because meat proteins at higher temperatures are more unstable than those at lower temperatures [9,24]. Therefore, preservation of meat at low temperatures is necessary to prevent drip loss-induced quality. The study showed that pork loin stored at supercooling temperatures reduces drip The changes in cooking loss for meat samples stored at different preservation treatments are shown in Figure 8B. The cooking loss of control samples was 26.84%, but that of the treated samples was 30.71%, 29.88%, 31.1%, and 30.6%. Overall, there was no significant difference in the cooking loss of pork loin after 8 days of storage, regardless of storage treatment (*p* > 0.05). However, the cooking yield of the treated samples was significantly higher than that of the fresh samples (*p* < 0.05).

Cooking loss is the weight loss caused by the release of liquid and hydrophilic substances during the cooking process [15]. Cooking causes protein denaturation and divests the protein structure of the trapped liquid due to the loss of capillary forces [25]. Chemically bound moisture in muscle meat is released through the process of protein degradation and fat melting during cooking [26]. Bentley et al. [27] observed that the cooking loss in refrigerated meat increased due to microbial proteolysis and protein denaturation, which resulted in plenty of gravy during cooking. Straadt et al. [28] observed an increased cooking loss in meat preserved at 4.0 °C for 4 days; the cooking loss was stable after this period for up to 2 weeks owing to the disintegration of injured fibers during aging. This study showed that cooking loss in treated samples was greater than that in fresh meat samples.

#### 3.4.3. TPA

**Figure 9:**
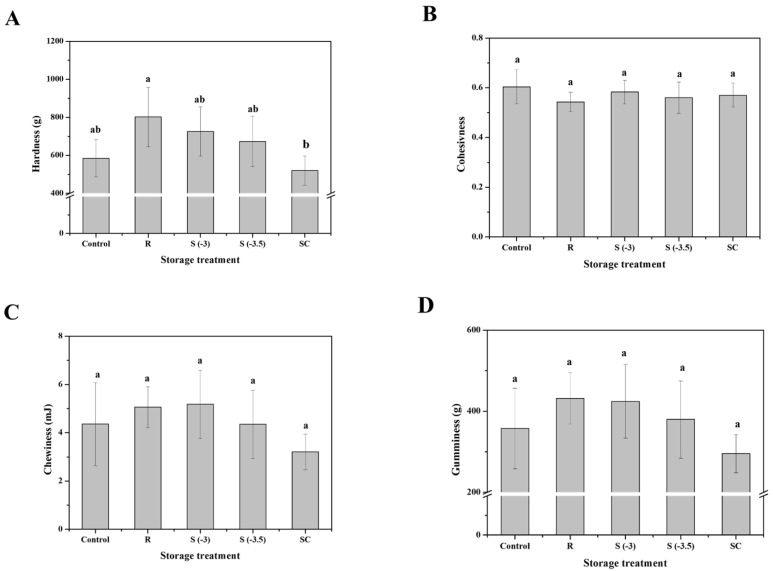
Changes in the (**A**) hardness, (**B**) cohesiveness, (**C**) chewiness, and (**D**) gumminess of pork loin with various preservation treatments. ^a, b^ Indicators using different letters are significantly different (*p* < 0.05).

The changes in the TPA values of pork loin samples preserved using various storage treatments are shown in Figure 9. The hardness of the fresh pork loin samples was 584.38 g, which was the lowest TPA value compared to that of the other treated samples, except for those that underwent freezing treatment (*p* < 0.05). However, the control samples were not significantly different from the supercooled samples (*p* < 0.05). Refrigerated treatment increased the hardness, chewiness, and gumminess values of the samples compared to the fresh samples, but there was no significant difference (*p* > 0.05). The cohesiveness of fresh meat was 0.60, and samples stored at refrigeration temperatures had the lowest TPA values. However, these samples did not show significant differences compared with the other treatments (*p* > 0.05). The chewiness value of fresh pork loin was 4.35 mJ, while that of the other treatments ranged from 3.20 to 5.2 mJ. Nevertheless, there were no significant differences in the chewiness values among the differently treated samples (*p* > 0.05). The gumminess of the control was 357.54 g, which increased under different storage treatments, except for freezing. In contrast, the gumminess of superchilled samples decreased to 295.55 g. Nevertheless, there were no significant differences among the various treatment groups (*p* > 0.05).

The texture of meat products is a crucial determinant of sensory quality and is influenced by several factors [29]. The tenderization of meat occurs through the destruction of muscle fibers and loss of structural integrity, which is a result of ice crystal formation in extracellular spaces [30]. Therefore, the hardness of the superchilled samples was lower than that of fresh meat. Vieira et al. [26] observed a decrease in the texture quality of meat samples stored under freezing conditions, which was consistent with the results of this study. Furthermore, an increase in the hardness value of refrigerated meat is mainly due to the degradation of proteins and muscle deterioration caused by the loss of the water-holding capacity of meat [31]. Therefore, supercooled storage is beneficial for delaying the degradation of proteins in pork loin compared to refrigerated storage.

#### 3.4.4. Color

**Figure 10:**
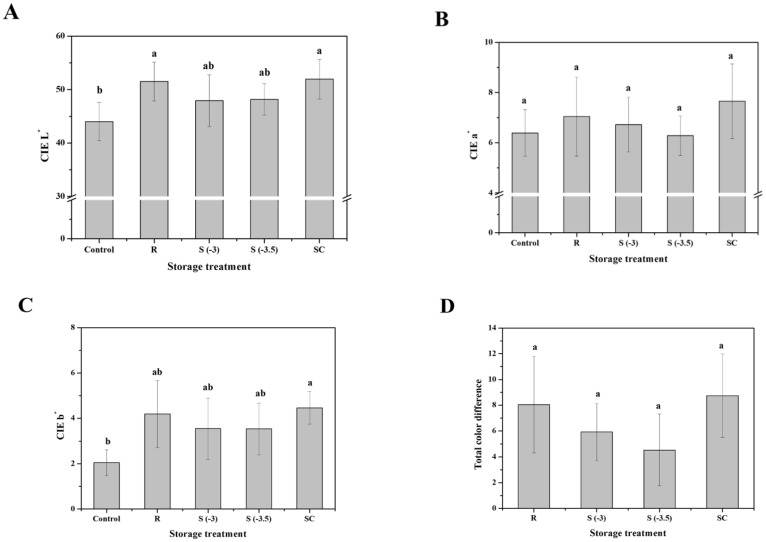
Changes in the (**A**) CIE L*, (**B**) CIE a*, (**C**) CIE b*, and (**D**) total color difference of pork loin with various storage treatments. ^a, b^ Indicators using different letters are significantly different (*p* < 0.05).

**Figure 11:**
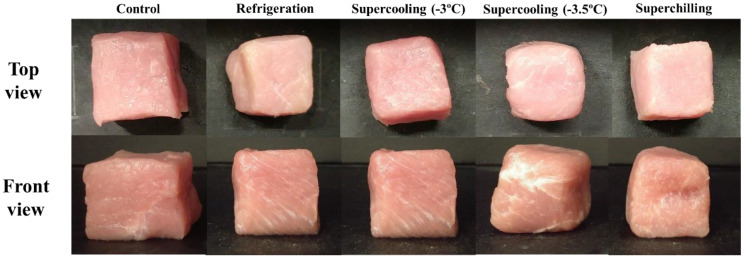
Changes in the appearance of pork loin with various storage treatments.

The color of pork loin after various storage treatments was determined and is presented in Figure 10. The CIE L* content of the control was 44, and preservation temperature had no effect on the CIE L*. After 8 days of storage, the CIE L* values of refrigerated and superchilled meat increased to approximately 52 and were significantly higher than those of fresh samples (*p* < 0.05). According to Lin et al. [32], an increase in the CIE L* value is caused by a change in the refractive index due to drip exudation. However, supercooled samples presented a slight increase in the lightness (approximately 48) of pork loin after eight days, which was not significantly different from that of fresh meat (*p* < 0.05). The CIE a* value of fresh pork loin was 6.39, and that value hardly changed during storage. The values of all treated samples increased compared to those of the control but did not significantly differ (*p* > 0.05). The CIE b* values were significantly increased after different storage treatments (*p* < 0.05). The CIE b* value of fresh pork loin was 2.05 and increased to 3.53–4.46 after the various storage treatments. However, no significant differences were observed between the treatment groups (*p* > 0.05). Similarly, the total color differences of the meat samples were diverse. However, there was no significant difference among the storage treatment samples after eight days of storage (*p* > 0.05). The refrigerated meat was slightly discolored, but the color of the other treatments was similar to that of the control (Figure 11). 

Meat color is recognized as a quality parameter; bright red is considered an indicator of freshness by consumers [33]. The storage temperature of meat is associated with its color, and high temperatures accelerate its discoloration rate [16]. Therefore, low temperature storage is important to decrease changes in the color of fresh meat [34]. Coombs et al. [35] found that supercooling storage efficiently maintained the color of fresh meat products, resulting from higher oxymyoglobin values, which equates to lower metmyoglobin values compared to samples stored at 2.0 and 5.0 °C [36]. In addition, Choe et al. [37] observed a greater change in the color of meat stored under chilling conditions than in samples stored at lower temperatures. This study demonstrates that supercooled storage maintained the color of fresh pork loins better than the other treatments. Preservation at supercooling temperatures was found to be more beneficial for delaying the discoloration of pork loin than freezing and refrigeration treatments.

#### 3.4.5. TVBN and Microbial Activity

**Figure 12:**
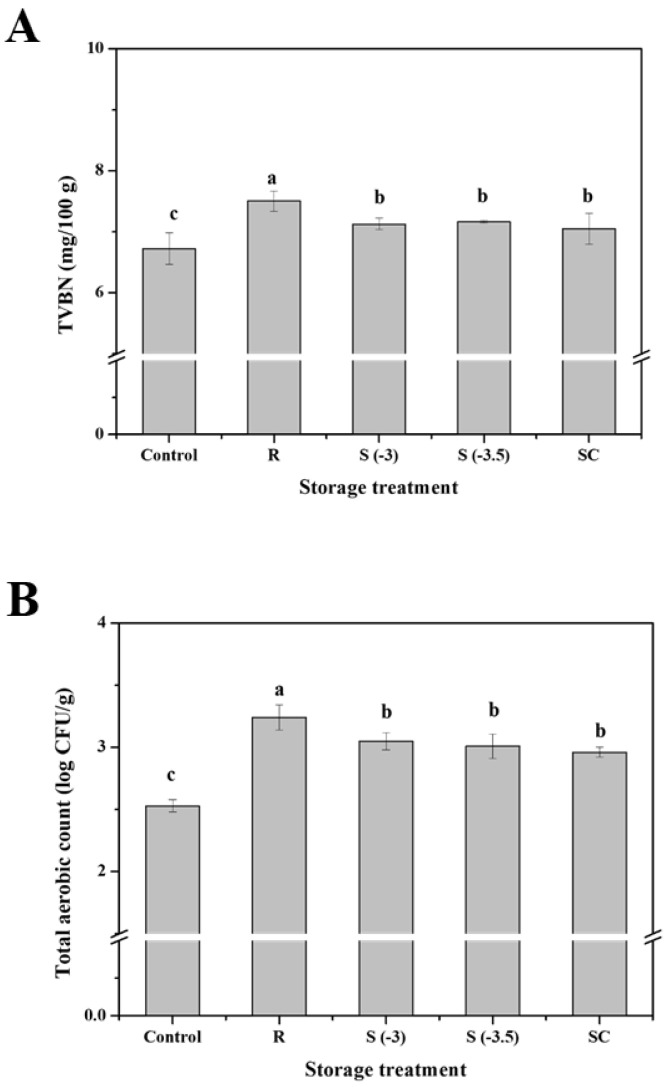
Changes in the (**A**) total volatile basic nitrogen (TVBN) and (**B**) total aerobic count (TAC) of pork loin with various preservation treatments. ^a–c^ Indicators using different letters are significantly different (*p* < 0.05).

The change in the TVBN values during various storage treatments of pork loin was determined. As presented at Figure 12A, the values of TVBN were influenced by the preservation temperature of the samples. The TVBN of the fresh sample was 6.72 mg/100 g, which increased at the end of the storage period. The TVBN value of the refrigerated sample was 7.50 mg/100 g, which was the highest among all treatment groups (*p* < 0.05). The TVBN values of the supercooled samples were 7.13 mg/100 g and 7.16 mg/100 g, which were lower than those of the refrigerated samples (*p* < 0.05). The TVBN of the superchilled samples was 7.05 mg/100 g, which is not significantly different from that of the supercooled samples (*p* > 0.05).

TVBN quantifies the presence of nitrogenous compounds such as amines and ammonia produced by protein degradation in meat and is considered a parameter of freshness [16,38]. These nitrogen-containing compounds are generated by proteolytic microbial activity [39,40]. Low temperature storage decreases TVBN production through the inhibition of metabolism [41]. The TVBN values of pork loin stored under superchilling conditions (−2.0 and −3.0 °C) were lower than those of meat stored at −1.0 °C [39]. Furthermore, Liu et al. [42] observed that grass carp fillets preserved at refrigeration temperatures had higher TVBN values than those preserved using other treatments. Results of our study indicate that supercooling effectively delays pork loin protein degradation compared to refrigeration.

Pork loin samples conserved using various storage treatments were examined for their total aerobic count (TAC), and the results are shown in Figure 12B. The TAC of the control sample was 2.53 log CFU/g. Overall, the TAC values of the treated samples depended on storage treatment. The TAC of the refrigerated samples was the highest among all storage treatment groups (*p* < 0.05). The TAC values of the refrigerated samples sharply increased to 3.24 log CFU/g after 8 days of storage. However, the TACs of the supercooled samples were 3.05 and 3.01 log CFU/g after 8 days of storage, which are significantly lower than that of the refrigerated samples (*p* < 0.05). The TAC of the superchilled samples was 2.96 log CFU/g, like that of the supercooled samples (*p* > 0.05).

Microbial activity is an important factor associated with the shelf life of meat products, and is influenced by temperature, pH, and water activity [43,44]. Microbial activity reduces macromolecules such as proteins into smaller molecules such as ammonia, amines, and hydrogen sulfide, causing off-odors [45]. Furthermore, microbial contamination results in off-flavor, slime formation, and discoloration [46,47]. Thus, low-temperature preservation is vital for controlling microbial growth and activity [32]. In this study, the overall tendency of TAC depending on the storage temperature was similar to that of the TVBN values. Refrigeration led to increased microbial growth in the pork loin owing to the higher preservation temperature, but this did not exceed the criterion for putridity over the storage period (6 log CFU/g) [16]. In comparison, the supercooling treatment inhibited microbial growth and activity, resulting in lower TACs, like those observed in the superchilled samples. Supercooling without phase transition hampers microbial growth and spoilage during storage and is beneficial for pork loin preservation.

## 4. Conclusions

This study was conducted to determine how external factors affect the maintenance of supercooling and pork loin freshness as preserved using various storage treatments. Through various experiments, it has been confirmed that there are three important factors for stable supercooling storage using static temperature control: (1) temperature deviation minimization, (2) setting the suitable lower-temperature limit, and (3) recovering the initial temperature of one cycle. Based on these results, an optimum stepwise cooling algorithm was designed for pork loin. Samples cooled to the lower-temperature limits of the different treatments using the designed algorithm were used to analyze the physicochemical properties of the treated samples. The pork loin samples conserved at −3.0 and −3.5 °C showed 100% and 92.6% efficiency at maintaining the supercooled state, respectively. The drip loss of the meat samples preserved using the supercooled treatment was lower than that of the samples stored using the superchilled treatment. The hardness, CIE L*, and CIE b* values of the samples conserved using supercooling treatments were significantly different from those of the fresh meat samples (*p* < 0.05). Furthermore, the TVBN and TAC of the supercooled storage samples were lower than those of refrigerated samples. Thus, supercooled storage of pork loins is beneficial for preventing quality degradation as compared with refrigerated storage. More importantly, external factors must be properly controlled to maintain the supercooled condition of food.

## Figures and Tables

**Table 1:** Experimental design for various slow cooling rates on pork loin.

Treatment	Initial Temperature (°C)	Precooling Time (h)	Time Interval (h)	Limit lower Temperature (°C)
A	−0.5	18	4	−3
B	−1	18	8	−3
C	−1	18	4	−3.5
D	−0.5	18	8	−3.5
E	−1	18	4	−4
F	−1	18	8	−4

## Data Availability

The data presented in this experiment are available on request from the corresponding author.

## Abbreviations

TVBN: total volatile basic nitrogen; TAC: total aerobic count; TPA: texture profile analysis; CIE: Commission Internationale de L’eclairage.
